# What Influences Patients' Adherence to Healthcare Worker Prescription in Primary Healthcare Facilities in Burkina Faso? A Qualitative Account of Barriers and Facilitators

**DOI:** 10.1093/cid/ciad347

**Published:** 2023-07-25

**Authors:** Adélaïde Compaoré, Jacqueline Nikièma, Francois Kiemdé, Halidou Tinto, Olawale Salami, Juvenal Nkeramahame, Piero Olliaro, Philip Horgan

**Affiliations:** Clinical Research Unit of Nanoro, Institut de Recherche en Sciences de la Santé, Nanoro, Burkina Faso; Clinical Research Unit of Nanoro, Institut de Recherche en Sciences de la Santé, Nanoro, Burkina Faso; Clinical Research Unit of Nanoro, Institut de Recherche en Sciences de la Santé, Nanoro, Burkina Faso; Clinical Research Unit of Nanoro, Institut de Recherche en Sciences de la Santé, Nanoro, Burkina Faso; FIND, Geneva, Switzerland; FIND, Geneva, Switzerland; FIND, Geneva, Switzerland; Evidence and Impact Oxford, Oxford, United Kingdom; FIND, Geneva, Switzerland; International Severe Acute Respiratory and Emerging Infection Consortium, Pandemic Sciences Institute University of Oxford, Oxford, United Kingdom; Nuffield Department of Medicine, Big Data Institute, University of Oxford, Oxford, United Kingdom

**Keywords:** adherence to prescription, ‌primary health care facility, qualitative research

## Abstract

**Background:**

This study explores the factors influencing patients and caregivers' adherence to prescription of healthcare workers (HCWs).

**Methods:**

The study was conducted in Temnaore and Pella, in the Nanoro health district in Burkina Faso. HCWs and community members were purposively recruited from 4 communities seeking care at the selected primary healthcare facilities for the clinical trial to attend in-depth interviews and focus group discussions on the factors influencing adherence to prescription. *The Behaviour Change Wheel* incorporating the Capability, Opportunity, and Motivation Behaviour approach was used.

**Results:**

Factors influencing the ability of patients to obtain the prescribed medicine include the availability of medicines and money and the perception of consequences for not getting the medicine. Regarding compliance with the intake of medicines, communication was considered a key factor whose effectiveness depends on the performance of HCWs and on the attention of patients. It is followed by other factors such as adequate management of patients, social influences, the patient’s beliefs regarding treatment, and memory.

**Conclusions:**

This research highlights factors influencing adherence to HCWs’ prescription from the perspective of the community members and HCWs and therefore provides contextual enablers and barriers, which allows for the development of an intervention to support the clinical trial.

## Teaser

This qualitative research describes factors influencing adherence to healthcare workers’ prescription from the perspective of users. Findings were used to inform training and communication interventions embedded in the Antimicrobial Resistance Diagnostics Use Accelerator clinical trial in Burkina Faso.

In resource-limited settings, fever is the major symptom reported for medical care [1]. Although malaria is recognized as the predominant febrile disease, fever could be attributed to other pathogens such as bacteria and viruses [2]. However, due to the lack of capacity, healthcare professionals sometimes face challenges in the management of these pathologies, especially in diagnostics and the administration of correct treatments. Deficiencies often lie in the limited material and human resources, diagnostic tests, drug supplies, and service management capacities [3].

Consequently, in malaria-endemic areas such as Burkina Faso, most fever cases have historically been treated as malaria [4–6]. In addition, with the emergence of bacterial and viral infections, some febrile illnesses are systematically treated with antibiotics and or antimalarials [6]. This could be explained by the uncertainty of the diagnostic and the desire to not miss the opportunity to treat potential nonmalarial infections. Consequently, there is overuse or misuse of both antimalarial and antibiotics, contributing to the emergence of antimicrobial resistance (AMR), which can therefore affect the effective management of fever, often leading to patient referral to other health centers, long stay in hospitals, additional costs, and even poorer clinical outcomes [7–9].

Behavioral factors, such as healthcare workers’ (HCWs) prescribing behavior [10] and patients’ adherence as well as structural factors, are among other factors contributing to suboptimal health outcomes and the emergence of AMR. HCWs must use available and appropriate diagnostics and effectively communicate prescriptions to patients and caregivers. Patients and caregivers must obtain the medicines prescribed and use them as intended.

To overcome this, FIND, launched the AMR Diagnostic Use Accelerator project (ClinicalTrials.gov identifier NCT04081051) [11] at the AMR Call to Action event in Ghana on 18 November 2018. In this project, point-of-care tests and diagnostic algorithms were introduced in peripheral healthcare facilities to address the following question: Will the use of a package of available diagnostic tests, diagnostic algorithms, new clinic process flows, and training and communication for HCWs and patients or caregivers (intervention) improve case management of acute febrile illnesses in children, adolescents and adults who present to outpatient clinics or peripheral health centers in low- and middle-income countries (LMICs), and better target the correct use of antibiotics (outcome) compared to current practice (control)?

However, the effectiveness of such an intervention is highly tied to uptake of the proposed intervention by patients/caregivers and healthcare professionals. In addition, given the need to change patients’/caregivers' health-seeking behavior, especially their adherence to the prescription, it is essential that researchers develop a deep understanding of the social and contextual bottlenecks that act on them [12]. Therefore, qualitative research was designed to support this.

Qualitative research can potentially add value to clinical trials as it provides explanation to research bias, efficiency, ethics, implementation, interpretation, relevance, success, and validity [13, 14].

In this clinical trial, qualitative methods and behavioral frameworks were used to investigate 2 key behaviors—(1) adherence to prescriptions by patients and caregivers and (2) communication of adherence messages by HCWs—to answer the overarching research questions: (1) What are the behavioral determinants for adherence to prescriptions by patients/caregivers who present with fever symptoms at outpatient clinics in LMICs? (2) What are the behavioral determinants of communication of adherence messages by HCWs to patients/caregivers who present with fever symptoms at outpatient clinics of the selected LMICs?

This study describes factors influencing adherence to prescription by patients and caregivers, informed by the Capability, Opportunity, and Motivation Behaviour (COM-B) framework and the Theoretical Domain Framework (TDF), to develop the training and communication intervention. Prescription adherence is defined as obtaining (buying or being given) the prescribed medicine and taking that medicine following the prescription instructions of dosage, frequency, and duration.

## METHODS

### Study Site and Population

The study was conducted in the Nanoro health district in Burkina Faso. Nanoro is in the Centre-West region of the country, approximately 90 km west from the capital Ouagadougou and 75 km from the provincial and regional capital, Koudougou. It is a poor, rural region with a population estimated at 171 119 inhabitants in 2017 [15]. The literacy rate is approximatively 23% for both men and women [16].

Nanoro health district has 19 peripheral health centers and 1 rural missionary referral hospital (Centre Médical avec Antenne Chirurgicale, Saint Camille). In principle, people living in the area should have access to healthcare: Approximately 65% of the population lives within 5 km, 25% within 5–10 km, and 10% more than 10 km away from a primary health facility (Centre de Santé et de Promotion Social). However, the region is impoverished, with 53%–57% of people living on <0.7 US dollars per day [17], which contributes to self-medication practices. The Clinical Research Unit of Nanoro collates Health and Demographic Surveillance System data of the area [18].

In Nanoro, diseases caused by inadequate potable drinking water facilities and poor sanitation are the main causes of health problems [19]. Communicable diseases such as malaria, acute respiratory infections, and undiagnosed fever are leading causes of death among adults and under-5 children [10, 11]. Since 2010, rapid diagnostic tests for malaria have been made available in primary health centers by the Ministry of Health, but microbiology laboratory services are not available and thus do not form part of routine medical practice [20]. Instead, the Ministry of Health issued treatment guidelines including guidance on antibiotic treatment for children <5 years old [21].

### Research Design Theoretical Framework

Our research approach broadly falls within the subtle realism [22] school of thought reflecting that “an external reality exists but is only known through the human mind and socially constructed meaning” [23].

We use an interpretivist paradigm to “explore and understand the social world through the participants and [our] own perspective”’ [23] using qualitative methods that allow us to explore culturally specific values, behaviors, opinions, relationships, and social contexts of populations.

Qualitative research can potentially add value to clinical trials as it provides an explanation to research bias, efficiency, ethics, implementation, interpretation, relevance, success, and validity [13, 14].

We have taken a pragmatic epistemology approach, loosely using the framing of selected behavioral frameworks to inform guides for data collection and in shaping an analysis framework from which themes are inducted.

In this study, qualitative methods (in-depth interviews [IDIs] and focus group discussions [FGDs]) and behavioral frameworks are used to investigate the 2 key behaviors—(1) adherence to prescriptions by patients and caregivers and (2) communication of adherence messages by HCWs—to answer the overarching research questions: (1) What are the behavioral determinants for adherence to prescriptions by patients/caregivers who present with fever symptoms at outpatient clinics in LMICs? (2) What are the behavioral determinants of communication of adherence messages by HCWs to patients/caregivers who present with fever symptoms in outpatient clinics of the selected LMICs?

*The Behaviour Change Wheel* (BCW) guide [18, 24] incorporating the COM-B and TDF approaches is a guide to the structured analysis of behaviors and provides a framework to support the development of behavior change interventions.

The BCW and COM-B/TDF approaches were selected for use in the study following a short scoping review of behavior change frameworks (see Discussion) and used to help to design topic guides and the development of the training and communication package. The BCW, incorporating COM-B and TDF, was chosen over the other frameworks because of its breadth across environment and contextual factors and the wide-ranging review and consolidation process underpinning the TDF [25].

### Data Collection

The data collection was performed by 2 experienced social scientists with a background in sociology. Most of the interviews were conducted in the local language (Mooré) except interviews with the HCWs, which were conducted in French. The 2 focus groups also occurred in the local language. Flexibility was built into the topic guide to allow for some probing where it was required for clarity.

The design of research topic guides for IDIs and FDGs was influenced by the TDF and COM-B frameworks described in the BCW guide.

The topic guides include barriers and enabling factors to (1) the effective communication of HCWs with patients/caregivers about adherence to the prescription and (2) obtainment of and adherence to prescribed medicine by patients and caregivers.

The interview prompts have been formulated such that they cover the TDF areas of knowledge, skills, belief about capabilities, memory, attention and decision processes, physical opportunity, social influences/professional identity, belief about consequences, routines, and habits.

The individual interviews ranged from 25 to 60 minutes in duration and were conducted at locations chosen by the interviewees to facilitate their comfort and ease of expression. Most interviews were held in the vicinity of the health center, but at a reasonable distance to avoid any potential overhearing or disruption. Four interviews were conducted at the participants' homes.

The group discussions were conducted at the health centers and lasted 2 hours. To ensure thematic saturation, follow-up questions were used to explore each theme, and all participants were given an opportunity to share their views and provide multidimensional responses. The interviews and focus group discussions were recorded after permission was obtained from interviewees. All participants were informed about the purpose, risks, benefits, and procedures of the study before they decided to participate. They were also made aware of their right to withdraw from the interview or discussion at any time. Written consent was obtained from participants.

### Research Participants and Sampling Strategy

Patients and caregivers were recruited based on the health registry, which indicated individuals who visited the health center in the last 5 days due to fever or who accompanied and cared for a patient who visited the health center for fever-related reasons. The healthcare providers were nurses who assessed fever cases and prescribed medication. As for the participants in focus groups and potential patients, they are members of the community who seek medical care at the healthcare center involved in the study. Their recruitment was facilitated by field workers. Participants were purposively selected from 4 selected communities within each of the primary healthcare facilities.

Sixteen individuals participated in the 2 FGDs. FGDs were conducted with men and women in 2 separate groups of 8 participants. They were all farmers, the majority practiced income-generative activity as source of income (11 [68.7%]) and half had not attended school (Table 1). IDIs were conducted with 6 HCWs (representing all HCWs in the study health center (Table 2) and a further 15 community members; most of them were married and the majority had not attended school. They were mostly farmers, and the majority did not have any source of income (Table 3).

**Table 1.** Characteristics of Participants in the Focus Group (n = 16)

Characteristic	No. (%)
Sex	
Male	8 (50.0)
Female	8 (50.0)
Education	
None	8 (50.0)
Primary	2 (12.5)
Secondary school	2 (12.5)
None	2 (12.5)
Primary	1 (6.2)
Nonformal education	1 (6.2)
Source of income	
Income-generating activity	11 (68.7)
Community health worker	2 (12.5)
None	3 (18.7)

**Table 2.** Characteristics of Healthcare Workers (n = 6)

Characteristic	No. (%)
Role	
Nurse	5 (83.3)
Midwife	1 (16.7)
Sex	
Female	4 (66.7)
Male	2 (33.3)

**Table 3.** Characteristics of Community Members Interviewed (n = 15)

Characteristic	No. (%)
Inclusion criteria	
Caregiver	5 (33.3)
Community key informant	5 (33.3)
Patient	5 (33.3)
Age, y, mean	50
Sex	
Male	8 (52.4)
Female	7 (46.6)
Marital status	
Married/monogamy	7 (46.7)
Married/polygamy	6 (40.0)
Widow	1 (6.7)
Single	1 (6.7)
Education	
None	8 (53.3)
Secondary	3 (20.0)
Primary	3 (20.0)
Nonformal education	1 (6.7)
Source of income	
None	9 (60.0)
Income-generating activity	4 (26.7)
Sewing	1 (6.7)
Mechanic	1 (6.7)
Occupation	
Farming	12 (80.0)
Merchant	2 (13.3)
Student	1 (6.7)

During a preanalysis stage, we identified that at the end of data collection described above, the last responses provided no new themes and therefore concluded that data saturation had been reached.

### Data Processing

The collected data were first anonymized, and quotes to illustrate research findings are not identifiable by personal characteristics. The audio recording was renamed and simultaneously translated and integrally transcribed verbatim from the local language into French by 2 field social scientists. Files were password protected and accessible to the 2 social scientists who collected the data, the site social sciences lead, and the work package lead.

### Analysis

The collected data were subject to framework analysis. A coding frame was first developed on NVivo version 12, a qualitative analysis software reflecting the topics' questions and prompts (psychological capacity [knowledge, skills, memory attention, decision processes]; physical opportunity; reflective motivation [belief about capabilities, social influences/professional identify, belief about consequences]; and automatic motivations [routines and habits]) (Supplementary Table 1).

In the topic guide, key themes were identified and grouped into the 2 main components of adherence to treatment and the purchase of the prescribed drug/taking the drug as *per* the prescription.

The preliminary coding was performed by the 2 field social scientists who collected the data. For consistency purposes of key themes, an independent researcher checked and resolved any discrepancies between the 2 researchers.

### Ethics Considerations

This study was approved by the Comité d’Ethique Institutionel pour la recherche en Sciences de la Santé (CEIRES) in Burkina Faso (reference number A09-2019/CEIRES) and the Comité d’Ethique pour la Recherche en Santé in Burkina Faso (reference number 2020-01-010) and received ethical approval as part of the approval of the overall trial protocol by the Oxford University clinical trials research ethics committee (OxTREC number 52-19).

Prior to the start of the interviews, all interviewees were informed about the project goals, topics, and type of questions as well as their right to decline participation or to interrupt the conversation at any time.

Locations for FGDs and IDIs were selected to provide confidentiality in data gathering. All personal identifiable information was removed from participant responses while transcribing the audio recordings and quotes to illustrate research findings.

## RESULTS

Participants reported several factors that influence adherence to prescription. These factors are presented as barriers and enablers under the 2 main domains of adherence (the purchase of medicine and the uptake of medicine) and described below with details and illustrated by verbatim quotes from IDIs and FGDs.

### Barriers to the Purchase of the Prescribed Medicine

#### Money Issues

In response to the question of whether money can make the purchase of the product more difficult, some people thought that money could not prevent them from buying the medicine.

*When you are ill, you cannot claim you do not have any money. You must manage to find money. (FGD with women)*

While some people’s attitudes are regarded as reckless or imprudent, financial issues are often involved.

*Someone can delay going to the hospital and people will think that he is joking with his health, but it is because he doesn’t have money. (FGD with women)*

The lack of money has implications for the failure to purchase the prescribed medicines, in which case some patients may resort to other, nonrecommended medicines. These often include “street medicines” (from informal drug sellers).

*Because you have no money to go there and because the disease makes you lose weight and you stand there, you buy the medicines at the market, and you see that the individual is not healing. By the time they get to the clinic, they are in a critical condition. (IDI with community key informant)*

#### Drug Shortages at Healthcare Facilities

Respondents reported that the reason why they do not adhere to prescription is because sometimes they struggle to buy treatment. This fact is worsened in certain conditions. For instance, local stockouts at the health facility drugstores are problematic. Failure to obtain the prescribed medicine at the healthcare facility leads patients to look for other alternatives to access it. This means they must buy them at private drugstores in the community, which implies extra expenses, and some budget is needed for transport to go to drugstores, which are sometimes in remote places. Furthermore, the medicine available at the healthcare centers is cheaper than that purchased from private stores. Consequences of the lack of money to buy medicine lead patients buy alternative drugs or use herbal medicine.

*You can come to the health facility with the child, and they give you the prescription. You go to the pharmaceutical depot and the drug is not available. It's available somewhere else but it is far. You can have the money to buy the drug but how will you manage to get there. (FGD with women)*

Drug shortage raises the issue of affordability, as the internal initiatives to make drug available pose a burden on the patient's pocket. In fact, to overcome drug shortage, the nurses buy medicines themselves and sell them to patients while they are waiting for the government to provide it.

*There may be times when the drugs are missing at the pharmacy and in this situation, the nurses consult each other, and they take the revenue of the care or the consultation to buy medicines to help the patients. Then the drugs become expensive because they are going to be looking for an interest in these drugs. (IDI with community key informant)*

#### Peers Providing Wrong Advice

Some people can negatively influence others, and this happens especially when the patient cannot afford the treatment cost and is obliged to rely on advice from others. In fact, some community members “mislead patients,” especially by telling patients that the prescribed medicine is not needed when they ask to borrow money. In addition, according to HCWs, some people, such as seniors, are considered as the main advisors who have more influence in the community, when it concerns child health.

*It is because he didn’t have money that he came home to look for it. They tell you it is not a disease for the hospital, why did you go there? Some people, when the drug is expensive, will ask for money everywhere and this is what brings this kind of words. Otherwise, if you had the money in your hands, you would buy it (IDI with a community key informant).*

*There are the old women at home who say that the medicines that were prescribed are too many. They say that if the child takes it, it will be too much for him and so on … (IDI with an HCW)*

### Enablers of the Purchase of the Prescribed Medicine

#### Availability of Financial Resources

According to some respondents, money is the most important factor that can facilitate the procurement of medicines for various reasons. If you have money, you can go to the hospital and purchase the medicines promptly. This will spare you the trouble of having to walk around to find cash. In addition, whatever the medicine costs in term of distance and location, the availability of resources makes it easier.

*What can facilitate the purchase of the medicine is the availability at the healthcare center. If we can buy the medicine locally it is easy for us. In this case, even if you don’t have enough money, there is no need for you to buy fuel to go and buy the medicine somewhere else. (IDI with a patient)*

From the respondents' perspectives, their money refers not only to cash but also to the goods (crops, animals) that people can sell to solve any problem they face.

*What can also help is if you have something to sell, you can tell someone to take this goat or that thing, sell it for you to treat the ill person. This is what can facilitate. (IDI with a caregiver)*

#### Awareness of the Consequence of Not Purchasing the Right Medicine

Most of the respondents agreed on the consequences of buying a drug that was not prescribed. According to them, purchasing another drug can worsen illness. It can also make you contract other illnesses. And worse, you can die.

*The disease can grow worse. If they show you how you should do, and you take it as you want or you don’t take time to know whether the medicine is for the head, the tummy, or the leg. Or they prescribe for you a medicine for the stomach, and you buy a medicine for headaches or for legs, it can bring other diseases. (FGD with men)*

#### Timely Presentation at the Healthcare Facilities and Costs

According to respondents, prompt presentation at the healthcare center can facilitate their adherence to the prescription. In fact, seeking care at the clinic at the onset of the illness is likely to have an impact on the price of the treatments. Since the illness is at its early stage, it is mild and easy to handle with few financial resources. For instance, while the illness is at its early stage, it can be treated with tablets and therefore avoids the need for expensive treatment such as intravenous injections.

*If the disease starts and you bring the child to the hospital quickly, it can be easy. But if you stay home pretending to have things to do, if you let the disease grow worse before going to the healthcare center, the purchase of the drug can be difficult because the medicine and the disease are proportional. (FGD with men)*

Participants in the interviews do know that delaying the treatment course can be detrimental to the illness's outcome. However, they claim this situation is rooted in financial issues. Being faced with lack of money and the mechanisms developed to sort it out, patients/caregivers are inclined to receive care late.

*When someone is not in a hurry to go to the clinic it is because of lack of money. If the child comes back from his games and tells you that he is unwell, you make a calculation and you have nothing in the house to take him to the hospital; you can go to borrow the money from someone, and that person will tell you that he doesn’t have. It can delay coming to the healthcare center. (FGD with women)*

#### Solidarity and Compassion

Solidarity is a factor that can facilitate the purchase of the drug according to some people. Indeed, you can be short of money but if in your surroundings there are people who can support you, that can be helpful to getting the drug.

*When the child is sick, you go home, you turn around in the house and you don’t have money to buy the medicine, it is difficult for you. But if you get help you will buy the medicines quickly and this really takes a load off your mind. (FGD with women)*

There are opportunities offered by drugstores to enable patients to obtain the medicines prescribed so that they can complete their treatment.

*If you go at the pharmacy with the prescription that you were given by nurse, if you don’t have enough money, the pharmacist will give you half of the medicines and keep the prescription. Then he will tell you to go and take what he gave you today, tomorrow and come back two days later to buy the rest of the medicines … This healthcare center helps people by giving them half of the medicines for you to come three days later to buy the rest and complete the treatment. (IDI community key informant)*

#### The National Policy for the Health of Children Under 5

The government of Burkina Faso has adopted a law stating that children aged <5 years can receive medical care, free of charge. For some people, this makes it easier to access medicine because they do not need to spend money. This was underlined by one respondent.

*What makes it easy for us to buy the drug is the fact that for children under five years old, the medicines are free. This is helpful for us. (FGD with women)*

### Barrier to Factors Influencing the Uptake of the Medicine as per Prescription

#### Poor Communication Between Patient and Healthcare Provider

Adherence to the uptake of medicine as instructed by the HCW is associated with patients’ and caregiver's ability to understand the prescription. It has been reported that some patients do not comply with the treatment regimen because of confusion about the prescribing details as communicated by the HCW. This means that the quality of communication during consultation does not allow patients to understand the prescription requirement. According to the respondents, failure related to the communication is due to various factors such as stress, the length of the consultation or patients' impatience, and HCWs’ lack of clarity to providing messages.

#### Lack of Clarity During Prescription

Some deficiencies were raised from the care providers' side as well. By way of example, it was noticed that HCWs do not provide enough details on whether the treatment should be taken until it finished.

*When the healthcare workers tell us to take the medicines, they remind us of this (they remind us to take the tablets) but even if you feel better, the medicine should not remain. This is what they don’t remind us. (IDI with a community key informant)*

Other factors affecting clarity of explanations include healthcare providers' inability to provide clear explanations in the language spoken by the patients.

*There are some healthcare workers who don’t speak well Mooré [local language]. If this kind of healthcare worker talks to you, if you don’t listen carefully, you will not understand. (FGD with women)*

This inability is accompanied by the provider’s inappropriate attitude toward patients.

*You can arrive at a healthcare center and they ask you: What is your problem? And at the same time, they start writing the prescription. When they ask you what your problem is, by the time you finish talking, the prescription is ready. (IDI with a community key informant)*

Moreover, communication issues are noticed especially in the context where patients are prescribed a variety of drugs in a way that patients are confused.

*They write two in the morning, two in the evening, one in the morning, and one in the evening. Like four drugs, they put them all together and they give it to you. Once you get home you cannot make it. (IDI with a community key informant)*

#### Patients’ Complaints About the Time of Consultation

In addition, according to healthcare providers, the conditions for them to communicate adequately require that patients cooperate and do not complain about the long waiting time, as this affects their ability to dedicate enough time to communicate with patients.

*More and more if there is a long queue, you will see people are going to start whispering, complaining because they think that a single person takes too much time whereas to do well the job there is a time given to each patient. But in our context, if we want to use this time, we will not be able to handle all the patients and that can also stir up other revolts from patients who are waiting. (IDI with HCW)*

#### Stress

The stress felt by patients or caregivers attending the healthcare facilities were reported to impede their listening capacity and means that they do not hear or internalize the information provided by the healthcare professional.

*This is because some are fearful. If he comes with the patient and the disease is serious, what they tell him to do … what you say, he hears but it doesn’t enter in his head (he doesn’t get it). He is scared. (IDI with HCW)*

#### Insufficient Human Resources and Time Available for Consultation

HCWs and community members highlighted that healthcare staff are overloaded, affecting patients’ adherence to prescription. While they are short in number, HCWs receive a huge number of patients for consultation per day. Consequently, they have limited time for consultation and inadequate time to communicate prescription adherence information. The priority for HCWs is to ensure that all patients are received.

*… But let's recognize that it is linked to the fact that we don’t have the accurate number to do the work as it should be done … when you do the ratio, you realize that we cannot. If you want to hang on about communication (which is important) it becomes difficult to handle all the patients who are waiting for us … if there is a long queue, you will see people are going to start whispering, complaining because they think that a single person takes too much time … if we want to use this time we will not be able to handle all the patients and that can also stir up other revolts from patients who are waiting. (IDI with HCW)*

#### Patient Belief and Treatment Preference

The perception that some types of drug delivery are better than others was highlighted. Some respondents expressed their preference for injections over tablets, which hindered adherence to a prescription consisting of tablets. On the other hand, others prefer tablets over injections, highlighting the issue of taste and difficulty to swallow, especially for children. The preference of injection-based treatment lies in its practicality to administer.

*What we can add is that when it is the injections, it is better. Some children don’t want the medicines at all. If you give it to him, he vomits. So, if we can have the injections, it is better. Some children do not like the taste of the drugs … because it is bitter. (FGD with women)*

#### Memory Issues

Patients' memory is essential for adherence to prescriptions. It intervenes by reminding when and how the drugs must be taken. Respondents reported that patients forget either to take their medicine or do not remember how and when to take them. Forgetfulness is reportedly caused by unexpected events in the neighborhood or family, or because of other activities such as working in the field, or because of old age.

*Yes! There are times when we forget because when you give it and the child starts to recover, with multiple occupations, you forget to give the medicine to the child. If you skip this day, the child will not recover. If you resume after this skipped day, you are making it worse. (IDI with a caregiver)*

*Old people cannot even take care of themselves. You can give them advice and try to get them to understand a situation, but they forget quickly. They no longer have memory. They mix things up. So, it doesn’t work often. **(**IDI with HCW)*

### Enablers of the Uptake of the Medicine as per Prescription

#### Effective Communication

According to an HCW, communication goes beyond the diagnostic and the prescription. The outcome of the illness depends on it as because without appropriate communication, it is impossible to guarantee patient recovery.

*First of all, between a patient and a healthcare worker if there is no communication, there is no care. Because you can do the diagnosis, conduct a good treatment but if there is not a good communication between your patient and yourself, you cannot have the desired result … So, I figure that communication is very fundamental between the healthcare provider and the patient. (IDI with HCW)*

For communication to be effective, the quality of human resources is essential. For instance, having enough well-trained healthcare workers can help communicate efficiently.

*I was talking about the healthcare workers facing the high number of patients. If we have more staff, it can be an advantage on the one hand and more if the agents are trained. It will improve the level of communication. (IDI with HCW)*

In a healthcare context, communication is necessary when a diagnosis must be made and is required all along the consultation.

*For me human activity is based on communication … to know what someone is suffering from, you should communicate. Even to help someone you should communicate. From his access to the healthcare center till he leaves, everything is based on communication. (IDI with HCW)*

It has also been reported that it is easier to communicate to the patient about the prescription when they believe that the patient can afford to buy the prescribed medicine.

*Yes, because if you are in front of someone who reassures you that he wants to buy the medicines, it can facilitate. Because it is frustrating for a healthcare worker to do a prescription with all the exams and think that this is … if you take it you will recover. Then you tell him without checking how much it is that you don’t have enough money. (IDI with HCW)*

Patient educational level is also relevant. HCWs find that patients who went to school are easy to interact with.

*Sometimes, if it is patients who are literate it is easier. (IDI with HCW)*

#### The Use of Triggers and Aids to Overcome the Issue of Memory

Community members described common strategies used to ensure that they recall the hours of medication intake as instructed by the HCW. This includes use of mobile phones, timing of the school bell, reminders from other people, and the position of the sun/shadows. For the same purpose, HCWs use triggers to help people remember when to take their medicines.

*… But nowadays, the mobile phones help a lot. Unless the phone isn’t on time, we look there to give the medicines. (FGD with men)*

#### Adequate Management of Patients’ Conditions

Accurate diagnosis and prescribing are both essential elements for adherence according to the respondents. Some respondents highlighted that having an accurate diagnosis from the HCW prevents them from buying unnecessary or ineffective drugs, which may affect their trust in the HCW prescription.

*If the healthcare worker knows exactly what the sick person is suffering from, it is then easy for us to buy the medicine. (FGD with women)*

However, according to HCWs, providing an accurate diagnosis and thus an appropriate treatment is limited by the lack of material, human resources, drug supplies, and service management capacities.

*Sometimes you are here, there are patients who are here; there are deliveries, ANCs [antenatal care], consultations … you want to handle patients and … in the ward there are women crying. So, you cannot take all your time to handle just one person. Sometimes this is what blocks us. If it was only consultations, you can take your time but if you are the one who does all the rest it is complicated. (IDI with HCW)*

#### The Dissuasive Power of the Father

To get treatment, children must rely on their parents. Consequently, their recovery is heavily tied to their parents' behaviors. According to the respondents, parents should be understanding and flattering toward their children to guarantee their recovery.

*To be able to give the drug as directed, you should cherish the child. Some children, if you pamper them, will take it well. If he doesn’t want to take and you threaten him by saying that if he doesn’t take it, you will leave him, it doesn’t encourage him to take. But if you flatter him, he will take the drug and he will recover. (FGD with men)*

Another strategy uses food to make the child take the drug.

*… you prepare for him a good dish that you don’t prepare often, and you tell him: take the tablet and then eat. Take the tablet and eat otherwise the others who are looking at it will come and take it. So, he takes it [laughs]. (FGD with women)*

Although mothers are considered the primary caregivers, there are some situations where the role of the “father as policeman” is requested.

*Fathers represent authority, which gives them the power to establish certain rules of conduct. Therefore, his role as a policeman is more impactful hence the necessity of their presence at the administration of drugs to the children. (FGD with men)*

## DISCUSSION

This research allows the identification of factors influencing patients’ or caregivers’ adherence to HCWs' prescription in primary healthcare facilities in Nanoro. In our study, adherence to prescription referred to the patient’s ability to purchase and uptake the medicines as recommended by the HCW. The findings highlighted that major factors influencing the purchase of medicines and their uptake are rooted in healthcare infrastructure organization. Some other factors were tied to patients' beliefs and perceptions.

### Factors Influencing the Purchase of Medicine

For treatment to be dispensed, drug must be affordable, available, and accessible to patients. In our study, drug availability and patient ability to pay for medicines were considered major factors influencing the purchase of medicines. On the one hand, the availability of drug was more challenging than the money issue. For instance, in Burkina Faso, although healthcare is free for under-5 children, caregivers sometimes struggled to find the appropriate treatment due to stockouts in public drugstores. On the other hand, the availability of money to pay for the treatment is constraining for people older than this age group. Stockouts and out-of-pocket for payment contribute modifying patients' care-seeking itinerary, which leads to increased healthcare cost for the patients. The downside of drug stockouts is that patients are compelled to rely on other drugs sold outside formal healthcare facilities, thereby resulting in suboptimal treatment, delayed care, referral to other facilities, and antimicrobial resistance.

However, access to medicine does not constitute sufficient element to guarantee adherence to healthcare prescription. Patients' uptake of medicine as indicated by the HCW was also regarded in our study.

### Factors Influencing the Uptake of Medicines

Regarding the uptake of medicine as recommended, our findings revealed that the adequacy and size of the health staff constitute important factors that impact on patient adherence. The adequacy and the size of the healthcare staff influence adherence at different levels.

### Communication Between Care Providers and Patients

Although communication was key to patients' ability to understand and remember prescriptions, its effectiveness was rooted in the quality of interaction between the patient and care provider. HCWs having good communication skills is essential to enable good adherence. It is necessary for providers to communicate perfectly so that the patient understands and does not forget, but it also requires the patient to be attentive and have the skills to capture and understand. For instance, studies have highlighted that although patients receive medication information and adherence advice, there is a lack of follow-up.

### Trust in the Healthcare System

In addition, the accurate diagnosis of illness and adequate prescription were considered facilitators of adherence. The lack of diagnostic and irrational prescribing is among the challenges that face primary healthcare facilities in LMICs such as Burkina Faso. Nevertheless, these elements are indicative of the competencies of the healthcare providers and therefore enforce patient trust in the healthcare system.

### Patients' Beliefs of Healthcare Influence Adherence

Our findings also highlight that the preference of the treatment is likely to affect patients’ motivation to adhere. Treatment preferences’ impact on adherence was largely studied from various perspectives. By the way of illustrations, quantitative research demonstrated that negative beliefs about medication have a greater impact on patients' adherence than other external factors [26, 27]. The treatment preference generally reflects patients’ perception of efficacy and safety. In our study, treatment efficacy was highlighted in terms of patients’ perception of its ability to cure quickly and its administration route. For instance, injections were requested because the mode of administration minimizes the loss of doses at administration, there is no need to worry about the taste, and it does not really engage the patient compared to tablets. In fact, physical barriers such as the size and the taste of the medication were among reasons for medication nonadherence, and this was shown in previous studies [28, 29].

Adherence to prescription is also influenced by patients' perceived benefits of healthcare. In our study, seeking care promptly was consider a factor that is likely to reduce healthcare cost burden on patients, hence allowing patient to purchase the recommended drugs.

The data have several limitations. First, the study reported on participants’ perspectives on how they engaged with prescriptions, rather than reporting on observed behaviors within community and households. Second, individuals’ social context and economic situation were not deeply explored.

## CONCLUSIONS

This research highlighted factors influencing adherence to HCWs’ prescription from the perspective of the community members and HCWs and therefore provided contextual enablers and barriers, which allows for the development of an intervention to support the clinical trial (Supplementary Table 2).

This study also showed adherence to be related to the shortcomings in the healthcare system, which could not be addressed in the clinical trials. However, these remain important findings reflecting on the challenges to ensure patients’ adherence to medication in resource-limited settings.

## Supplementary Data

Supplementary materials are available at *Clinical Infectious Diseases* online. Consisting of data provided by the authors to benefit the reader, the posted materials are not copyedited and are the sole responsibility of the authors, so questions or comments should be addressed to the corresponding author.

## Supplementary Material

ciad347_Supplementary_Data

## Notes

***Author Contributions*.** P. H. and A. C. designed the study. A. C. analyzed and drafted the manuscript. J. Ni. collected and analyzed the data. F. K., H. T., and J. Nk. revised the manuscript. P. H. supervised the study and revised the manuscript contents.

***Financial support***. This work was supported by the Swiss Agency for Development and Cooperation, the Department for International Development, United Kingdom, now the Foreign, Commonwealth and Development Office, United Kingdom and the Federal Ministry of Economic Cooperation and Development /Bundesministerium für Wirtschaftliche Zusammenarbeit und Entwicklung (BMZ).

***Supplement sponsorship*.** This article appears as part of the supplement “Using Diagnostic Tools to Support Antimicrobial Stewardship and Improve Outcome in Resource-Limited Contexts,” sponsored by FIND.
