# Supplementary material for: What Influences Patients' Adherence to Healthcare Worker Prescription in Primary Healthcare Facilities in Burkina Faso? A Qualitative Account of Barriers and Facilitators
Source: Clin Infect Dis. 2023 Jul 25;77(Suppl 2):S171–81. doi: 10.1093/cid/ciad347 (PMC10368408; doi:10.1093/cid/ciad347)
Supplement: ciad347_Supplementary_Data [file ciad347_supplementary_data.zip › Compaore et al_BF SocSci_Supplementary materials.docx]

# Supplementary materials

## Table 1. Codebook

| **Name** | **Description** |
| --- | --- |
| 1 Questions générales |  |
| 1.1 Facteurs favorables pour l’achat de médicament | Encoder à ce nœud toutes les parties de texte portant sur ce qui pourrait faciliter la tâche pour l’achat de médicament prescrit par le centre de santé. |
| 1.2 Facteurs favorables pour la prise du médicament | Encoder à ce nœud toutes les parties de texte traitant de ce qui pourrait faciliter la tâche pour prendre le médicament en respectant les instructions du centre de santé. |
| 1.3 Facteurs facilitant la prise du médicament par l'enfant | Encoder à ce nœud les parties de texte traitant de ce qui facilite la prise des médicaments par l’enfant selon les instructions du centre de santé |
| 1.4 Obstacles à l’achat le médicament | Encoder à ce nœud les parties de texte traitant de ce qui complique la tâche pour l’achat de médicaments prescrits au centre de santé |
| 1.5 Obstacles à la prise de médicament | Encoder à ce texte les parties de texte traitant de ce qui complique la tâche pour la prise le médicament en respectant les instructions du centre de santé |
| 1.6 Obstacles à la prise du médicament par l'enfant | Encoder à ce nœud les parties de texte traitant de ce qui complique la tâche pour s'assurer que l'enfant prenne le médicament en respectant les instructions du centre de santé. |
| 2. Connaissances et compétences/conviction en matière de capacités |  |
| 2.1 Perception des connaissances et compétences nécessaires | Encoder à ce nœud les parties de textes portant sur les connaissances et les compétences nécessaires pour le respect des instructions de la prescription. |
| 2.2 Les connaissances et compétences acquises | Encoder à ce nœud les parties de textes traitant des connaissances et compétences dont le répondant pense disposer. |
| 3. Mémoire, attention et processus de décision |  |
| 3.1 Prise de décision par rapport à la prise du médicament. | Encoder à ce nœud les parties de texte traitant de la décision du moment et de la façon de prendre le médicament |
| 3.2 Facteurs d'influence physique |  |
| 3.2.1 Influence facteur temps sur l'achat et l'utilisation | Encoder à ce noeud les parties de texte traitant de l'influence du temps sur l'achat du médicament prescrit et de son utilisation conformément aux instructions |
| 3.2.2 Influence facteur argent sur l'achat et l'utilisation | Encoder à ce noeud les parties de texte traitant de l'influence du facteur argent sur l'achat du médicament prescrit et de son utilisation conformément aux instructions. |
| 3.2.3 Influence facteur disponibilité sur l'achat et l'utilisation | Encoder à ce noeud les parties de texte traitant de l'influence de la disponibilité sur l'achat du médicament prescrit et de son utilisation conformément aux instructions |
| 3.2.4 Comment cela facilite la tâche | Encoder à ce noeud les parties de texte traitant des facteurs temps, argent et disponibilité qui favorisent l'achat du médicament prescrit et son utilisation conformément aux instructions, de ce qui facilite. |
| 3.2.5 . Comment cela complique la tache | Encoder à ce noeud les parties de texte traitant des facteurs temps, argent et disponibilité qui empêchent l'achat du médicament prescrit et son utilisation conformément aux instructions, de ce qui complique. |
| 3.3 Influence des attentes des autres, des familles | Encoder à ce noeud les parties de texte traitant des attentes des autres, des familles qui influencent la décision d'achat du médicament et son utilisation conformément aux instructions de l'établissement de santé. |
| 3.3.1 Impact positif de l’Influences des autres, des membres de la famille | Encoder à ce nœud les parties de texte traitant de l’influence des attentes des autres membres de la famille dans la communauté favorables à la décision d'achat du médicament et son utilisation conformément aux instructions du centre de santé |
| 3.3.2 Impact négatif de l’influence des membres de la famille | Encoder à ce nœud les parties de texte traitant de l’influence des attentes des autres membres de la famille dans la communauté qui affectent la décision d'achat du médicament et son utilisation conformément aux instructions du centre de santé |
| 3.4 Impression sur le non-achat du médicament | Encoder à ce nœud les parties de texte traitant de l'impression de quelqu'un qui n'achète pas le médicament prescrit auprès de l'établissement de santé |
| 3.5. Impression sur le non-respect des instructions | Encoder à ce nœud les partie de texte traitant de l'impression sur le non-respect des instructions de l'établissement de santé |
| 3.6 Perception des conséquences de l'achat d'un autre médicament | Encoder à ce nœud les parties de texte traitant des conséquences de l'achat d'un article à la place d'un autre |
| 3.7. Conséquence du non-respect des instructions | Encoder à ce nœud les parties de texte qui traitent des conséquences de prendre un médicament sans respecter les instructions |
| 3.8 Rôle des procédures et habitudes dans le choix du médicament | Encoder à ce nœud les parties de texte traitant du rôle des procédures et habitudes dans le choix du médicament acheter |
| 3.9 Proposition pour une bonne communication | Encoder à ce nœud les parties de texte traitant des propositions pour une bonne communication |
| 4. Rôle au sein du centre de santé | Encoder à ce nœud les parties de texte traitant du rôle des agents de santé au sein du centre de santé |
| 4.1 Communication patients et agents de santé | Encoder à ce nœud les parties de texte traitant de la communication entre les patients et les agents de santé |
| 4.2 Influence de la communication sur l'adhésion à la prescription | Encoder à ce nœud les parties de texte traitant de l'influence de la communication sur l'adhésion des patients à la prescription |
| 4.3 Faciliter la tâche de la communication pour l'adhésion | Encoder à ce nœud les parties de texte traitant de ce qui pourrait faciliter la tâche pour communiquer avec les patients accompagnants en ce qui concerne l'adhésion à la prescription |
| 4.4 Compliquer la tâche de la communication pour l'adhésion | Encoder à ce noeud les parties de texte qui traite de la complication de la tâche pour communiquer avec les patients et aidants en ce qui concerne l'adhésion à la prescription |
| 5.1 Connaissance et compétences pour communiquer | Encoder à ce noeud les parties de texte qui traitant des connaissance et compétences nécessaires pour communiquer avec les patients/aidants en ce qui concerne l'adhésion à la prescription |
| 5.1. Connaissances et compétences disposées | Encoder à ce noeud les parties de texte traitant des connaissances et compétences dont les agents de santé estiment disposer |
| 5.2 Décision de communication | Encoder à ce noeud les parties de texte traitant de la décision de ce qu'il convient de communiquer aux patients/aidants |
| 5.3 Les facteurs qui affectent la communication | Encoder à ce noeud les parties de texte traitant des facteurs qui affectent le contenu et la manière de communiquer avec les patients |
| 5.3.1 Facteur temps | Encoder à ce noeud les parties de texte traitant de l'influence du facteur temps sur le contenu et la manière de la communication |
| 5.3.2 Facteur disponibilité | Encoder à ce noeud les parties de texte traitant de l'influence du facteur disponibilité sur le contenu et la manière de communiquer |
| 5.3.3 Facteur argent | Encoder à ce noeud les parties de texte traitant de l'influence du facteur argent sur le continu et la manière de communiquer |
| 5.4 Facteur le plus déterminant | Encoder à ce noeud les parties de texte traitant du facteur le plus déterminant pour assurer une bonne communication |
| 5.4.1 Faciliter la tâche | Encoder à ce noeud les parties de texte traitant du facteur le plus déterminant pour assurer une bonne communication, comment cela facilite la tâche |
| 5.4.1 Compliquer la tâche | Encoder à ce noeud les parties de texte traitant du facteur le plus déterminant pour assurer une bonne communication, comment cela complique la tâche |
| 5.5 Les attentes des autres qui influencent la communication | Encoder à ce noeud les parties de texte traitant des attentes des autres dans la communauté qui influencent le contenu ou les modalités de la communication |
| 5.5. 1 Faciliter la tâche | Encoder à ce nœud les parties de texte traitant de la manière dont les attentes des autres dans la communauté influencent le contenu ou les modalités compliquent la tâche |
| 5.5.2 Compliquer la tâche | Encoder à ce nœud les parties de texte traitant de la manière dont les attentes des autres dans la communauté influencent le contenu ou les modalités complique la tâche |
| 5.6 Les attentes des autres professionnels médicaux | Encoder à ce nœud les parties de textes qui traitent des attentes des autres professionnels médicaux qui influencent le contenu ou les modalités de la communication |
| 5.6. 1. Comment cela facilite la tâche | Encoder à ce nœud les parties de texte qui traitent de ce qui pourrait faciliter la tâche par rapport aux attentes des autres professionnels médicaux qui influencent le contenu ou les modalités de la communication |
| 5.6. 1. Comment cela complique la tâche | Encoder à ce nœud les parties de texte qui traitent de ce qui pourrait compliquer la tâche par rapport aux attentes des autres professionnels médicaux qui influencent le contenu ou les modalités de la communication |
| 5.7 Impact de la communication | Encoder à ce nœud les parties de texte traitant de l'impact de la communication avec les patients /aidants en ce qui concerne l'adhésion à la prescription |
| 5.8 Rôle des procédures et habitudes dans la communication | Encoder à ce nœud les parties de texte traitant du rôle que jouent les procédures et habitudes dans la communication avec les patients/accompagnants en ce qui concerne l'adhésion à la prescription. |
| 5.8.1 Ce qui pourrait faciliter | Encoder à ce nœud les parties de texte qui pourraient faciliter le rôle que jouent les procédures et habitudes dans la communication avec les patients/ accompagnants en ce qui concerne l'adhésion à la prescription |
| 5.8.2Ce qui pourrait compliquer | Encoder à ce nœud les parties de texte qui pourraient compliquer le rôle que jouent les procédures et habitudes dans la communication avec les patients/ accompagnants en ce qui concerne l'adhésion à la prescription |
| 5.9 Proposition pour une bonne communication | Encoder à ce nœud les parties de texte traitant des propositions pour une bonne communication |

## Table 2: Support document of training and communication that was developed

|  | **Problèmes** | **Attitudes** |
| --- | --- | --- |
| **La consultation** | **La peur** | Rassurer les patients/accompagnants en leur disant que les agents de santé sont là pour eux. |
|  | **Le stress lié à l’état de santé d’un proche** | Aider l’accompagnant à comprendre que le patient a besoin de sa collaboration et de son courage pour se rétablir. |
|  | **Impatience des patients** | - Expliquer au patient que le temps passé avec l’infirmier est nécessaire pour l'aider à comprendre l'ordonnance, à suivre les instructions et à se rétablir.   - L’aider à comprendre qu’il n’est pas nécessaire de partir tôt s’il faut revenir au centre de santé pour le même mal. |
| **Les tests et rendus de résultats** |  | - Parler au patient de tous les types d'échantillons qui seront pris ;  - Rassurer le patient sur la nécessité de faire ces tests et sur la confidentialité   - Parler au patient des résultats possibles. |
| **Annonce du diagnostic (si des germes ont été retrouvés)** | **L’oubli** | - Demander au patient s'il veut impliquer quelqu'un d'autre pour les explications, puis donnez le diagnostic   - Donner toutes les explications possibles sur la ou les maladies (nom, mode transmission, évolution avec ou sans traitement…) |
|  | **Croient que certaines maladies ne se soignent pas au dispensaire** | - Aider le patient à comprendre que malgré la bonne volonté du guérisseur traditionnel pour le soigner, il ne dispose pas des tests ou des outils précis pour fournir un diagnostic fiable sur sa maladie |
|  | **Manque de confiance au diagnostic** | - Attirer l'attention du patient/accompagnant sur le fait que les infirmiers sont bien formés pour soigner. |
| **Annonce du diagnostic (si aucune maladie n’a été trouvée et aucun médicament n’est nécessaire)** |  | - Expliquer au patient pourquoi il n’a pas besoin de prendre des médicaments ;   - Montrer au patient en quoi prendre des médicaments est non nécessaire dans ce cas. |
| **La prescription** | L’argent | - Veiller à ce que le médicament prescrit soit le plus efficace et le moins cher possible ;  - Prescrire uniquement ce dont le patient a besoin après le diagnostic (médicaments recommandés par l'algorithme) ;  - Contrôler la disponibilité des médicaments au dépôt ;  - Rassurer l’accompagnant en lui disant que les soins sont gratuits pour les enfants de 6 à 59 mois et les femmes enceintes et en post-partum (42 jours) ;   - Convaincre le patient/l’accompagnant de la nécessité de commencer le traitement immédiatement et de le terminer ainsi que la nécessité d’acheter le médicament dans un dépôt pharmaceutique. ; |
|  |  | - , Problèmes, Attitudes - La consultation, La peur, Rassurer les patients/accompagnants en leur disant que les agents de santé sont là pour eux. - Le stress lié à l’état de santé d’un proche, Aider l’accompagnant à comprendre que le patient a besoin de sa collaboration et de son courage pour se rétablir. - Impatience des patients, - Expliquer au patient que le temps passé avec l’infirmier est nécessaire pour l'aider à comprendre l'ordonnance, à suivre les instructions et à se rétablir. L’aider à comprendre qu’il n’est pas nécessaire de partir tôt s’il faut revenir au centre de santé pour le même mal. - Les tests et rendus de résultats, , - Parler au patient de tous les types d'échantillons qui seront pris ;- Rassurer le patient sur la nécessité de faire ces tests et sur la confidentialité Parler au patient des résultats possibles. - Annonce du diagnostic (si des germes ont été retrouvés), L’oubli, - Demander au patient s'il veut impliquer quelqu'un d'autre pour les explications, puis donnez le diagnosticDonner toutes les explications possibles sur la ou les maladies (nom, mode transmission, évolution avec ou sans traitement…) - Croient que certaines maladies ne se soignent pas au dispensaire, Aider le patient à comprendre que malgré la bonne volonté du guérisseur traditionnel pour le soigner, il ne dispose pas des tests ou des outils précis pour fournir un diagnostic fiable sur sa maladie - Manque de confiance au diagnostic, Attirer l'attention du patient/accompagnant sur le fait que les infirmiers sont bien formés pour soigner. - Annonce du diagnostic (si aucune maladie n’a été trouvée et aucun médicament n’est nécessaire), , - Expliquer au patient pourquoi il n’a pas besoin de prendre des médicaments ;Montrer au patient en quoi prendre des médicaments est non nécessaire dans ce cas. - La prescription, L’argent, - Veiller à ce que le médicament prescrit soit le plus efficace et le moins cher possible ;- Prescrire uniquement ce dont le patient a besoin après le diagnostic (médicaments recommandés par l'algorithme) ;- Contrôler la disponibilité des médicaments au dépôt ;- Rassurer l’accompagnant en lui disant que les soins sont gratuits pour les enfants de 6 à 59 mois et les femmes enceintes et en post-partum (42 jours) ;Convaincre le patient/l’accompagnant de la nécessité de commencer le traitement immédiatement et de le terminer ainsi que la nécessité d’acheter le médicament dans un dépôt pharmaceutique. ; - Certaines personnes pensent que les injections guérissent mieux que les comprimés ;Certains pensent que les médicaments à bas prix ne sont pas efficaces ;, Expliquer et rassurer le patient que les comprimés seront efficaces pour sa maladie et l'aider à comprendre que les médicaments génériques ont les mêmes molécules que les médicaments de spécialité ; - Explication de la prescription, La barrière linguistique, Demander de l'aide à un autre infirmier ; - Manque de précision sur le traitement (nombre de jours) Multiplicité des médicaments , - Expliquer clairement et complètement la prescription (doses par prise, jour, nombre de jours)Aider les patients/accompagnants à comprendre la nécessité de prendre les médicaments en fonction du nombre de jours et de comprimés par dose et par jour - Incompréhension des instructions écrites , Fournir aux patients/accompagnants des signes pour les aider à comprendre les prescriptions - Arrêt du traitement en cas d’amélioration ou de non-amélioration , - Parler au patient des effets secondaires et lui donner des conseils pour les surmonter s'il y en a.Parler au patient/accompagnant de la nécessité de terminer le traitement même s'il se sent mieux - L’oubli dû aux voyages ou aux travaux champêtres ;, - Demander au patient/accompagnant d'identifier des personnes qui peuvent l'aider à se souvenir de prendre ou de donner le médicament à l'heure indiquée ;Demander au patient/à l'accompagnant de redire ce qu'on lui a dit - Sensibilité de la mère-Non-implication du père, Demander à l'accompagnant d'impliquer le mari dans l'administration du médicament ; - Le niveau d'éducation , - Demander au patient de faire le lien entre le médicament et ce qui se passe dans la communauté (le son de la cloche de l'école ou de l'église le matin, à midi et le soir); Utiliser un langage facile à comprendre et donner aux patients quelques signes pour les aider à comprendre les prescriptions; - Le genre du patient (timidité chez les femmes), Rassurer les patients/accompagnants que le personnel de santé est là pour eux. - La programmation du rendez-vous, La distance entre le domicile et le centre de santé , Parler au patient de l'importance du suivi ; - Demander au patient de commencer à identifier les moyens dont il a besoin pour venir au suivi depuis la première consultation. |
| **Explication de la prescription** | La barrière linguistique | - Demander de l'aide à un autre infirmier ; |
|  | Manque de précision sur le traitement (nombre de jours)    Multiplicité des médicaments | - Expliquer clairement et complètement la prescription (doses par prise, jour, nombre de jours)   - Aider les patients/accompagnants à comprendre la nécessité de prendre les médicaments en fonction du nombre de jours et de comprimés par dose et par jour |
|  | Incompréhension des instructions écrites | - Fournir aux patients/accompagnants des signes pour les aider à comprendre les prescriptions |
|  | Arrêt du traitement en cas d’amélioration ou de non-amélioration | - Parler au patient des effets secondaires et lui donner des conseils pour les surmonter s'il y en a.   - Parler au patient/accompagnant de la nécessité de terminer le traitement même s'il se sent mieux |
|  | L’oubli dû aux voyages ou aux travaux champêtres ; | - Demander au patient/accompagnant d'identifier des personnes qui peuvent l'aider à se souvenir de prendre ou de donner le médicament à l'heure indiquée ;   - Demander au patient/à l'accompagnant de redire ce qu'on lui a dit |
|  | Sensibilité de la mère-Non-implication du père | - Demander à l'accompagnant d'impliquer le mari dans l'administration du médicament ; |
|  | Le niveau d'éducation | - Demander au patient de faire le lien entre le médicament et ce qui se passe dans la communauté (le son de la cloche de l'école ou de l'église le matin, à midi et le soir) ;   - Utiliser un langage facile à comprendre et donner aux patients quelques signes pour les aider à comprendre les prescriptions ; |
|  | Le genre du patient (timidité chez les femmes) | - Rassurer les patients/accompagnants que le personnel de santé est là pour eux. |
| **La programmation du rendez-vous** | La distance entre le domicile et le centre de santé | - Parler au patient de l'importance du suivi ; |
|  |  | - Demander au patient de commencer à identifier les moyens dont il a besoin pour venir au suivi depuis la première consultation. |
