# Supplementary material for: What Influences Patients' Adherence to Healthcare Worker Prescription in Primary Healthcare Facilities in Burkina Faso? A Qualitative Account of Barriers and Facilitators
Source: Clin Infect Dis. 2023 Jul 25;77(Suppl 2):S171–81. doi: 10.1093/cid/ciad347 (PMC10368408; doi:10.1093/cid/ciad347)
Supplement: ciad347_Supplementary_Data [file ciad347_supplementary_data.zip › Supplemental data-English version.docx]

1. Table 1. Codebook

| Name | Description |
| --- | --- |
| 1 General questions |  |
| 1.1 Favorable factors for the purchase of drugs | Encode at this node all the parts of text about what would make it easier to buy the medication prescribed by the health center. |
| 1.2 Favorable factors to taking the drug | Encode at this node all the parts of the text that deal with what would make it easier to take the medication according to the health care worker’s instructions. |
| 1.3 Factors that make it easier for the child to take the medication | Encode at this node the parts of the text that deal with what makes it easier for the child to take the medication according to the health care worker's instructions. |
| 1.4 Barriers to purchasing the drug | Encode at this node the parts of the text that deal with what makes it difficult to buy drugs prescribed at the health care center. |
| 1.5 Barriers to taking medication | Encode at this node the parts of text dealing with what complicates the task of taking the medication as directed at the health care center. |
| 1.6 Obstacles to the child taking the medication | Encode at this node the parts of the text that deal with what makes it difficult to ensure that the child takes the medication according to the health care worker's instructions. |
| 2. Knowledge and skills/belief in capabilities |  |
| 2.1 perception of the knowledge and skills required | Encode at this node the parts of the text that deal with the knowledge and skills needed to comply with the instructions of the prescription. |
| 2.2 Knowledge and skills acquired | Encode at this node the parts of texts dealing with the knowledge and skills that the respondent thinks he or she has. |

| Name | Description |
| --- | --- |
| 3. Memory, attention and decision making |  |
| 3.1 Decision making about taking the medication. | Encode at this node the parts of the text that deal with deciding when and how to take the medicine. |
| 3.2 Physical influencing factors |  |
| 3.2.1 Influence of the time factor on the purchase and use of the drug | Encode at this node the parts of the text dealing with the influence of time on the purchase of the prescribed drug and its use according to the instructions on the prescription. |
| 3.2.2 Influence of the money factor on purchase and use | Encode at this node the parts of the text dealing with the influence of the money factor on the purchase of the prescribed medication and its use according to the instructions. |
| 3.2.3 Influence of availability factor on purchase and use | Encode at this node the parts of the text dealing with the influence of the availability (of the drug) on the purchase of the prescribed drug and its use according to the instructions |
| 3.2.4 How do they (time money and availability) make it easier? | Encode at this node the parts of the text dealing with how time, money and availability factors facilitate the purchase of the prescribed medication and its use according to instructions. |
| 3.2.5. How do they (time money and availability) complicate it? | Encode at this node the parts of text dealing with how time, money and availability factors prevent the purchase of the prescribed medication and its use according to the instructions. |
| 3.3 Influence of the expectations of others, families | Encode at this node the parts of text dealing with how the expectations of others, of families influence the decision to purchase and use the drug according to the instructions given at the health care facility. |
| 3.3.1 Positive influences of others, family members | Encode at this node the parts of the text dealing with the influence of the expectations of others in the community or family members that are conducive to the decision to purchase the drug and use it according to the health center's instructions. |

| Name | Description |
| --- | --- |
| 3.3.2 Negative influence of family members | Encode at this node the portions of text dealing with the influence of expectations of other family members in the community on the decision to purchase the medication and its use according to the health center's instructions. |
| 3.4 Perceptions about not buying the drug | Encode at this node the parts of the text that deal with the perception of someone who does not purchase the medicine prescribed at the health care facility. |
| 3.5. Perception about the non-compliance with instructions | Encode to this node the parts of the text dealing with the perception about the non-compliance with the instructions given at the health care facility |
| 3.6 Perceived consequences of purchasing another drug | Encode at this node the parts of the text that deal with the consequences of buying a medicine instead of another. |
| 3.7. Consequence of not following the instructions | Encode at this node the parts of the text that deal with the consequences of taking a drug without following the instructions. |
| 3.8 Role of procedures and habits in drug selection | Encode at this node the parts of the text dealing with the role of procedures and habits in the choice of medication. |
| 3.9 Suggestion for a good communication | Encode at this node the parts of the text dealing with suggestions for good communication. |
| 4. Role within the health center | Encode to this node the parts of the text dealing with the role of the health care workers in the health center. |
| 4.1 Patient and health care worker communication | Encode at this node the parts of the text that deal with communication between patients and health care workers. |
| 4.2Influence of communication on adherence to prescription | Encode at this node the parts of the text dealing with the influence of communication on patient adherence to prescription. |
| 4.3 Factors that facilitate the communication for adherence to treatment | Encode at this node the parts of the text that deal with what would make it easier to communicate with patients and caregivers regarding adherence to the prescription. |

| Name | Description |
| --- | --- |
| 4.4 Factors that complicate the communication for adherence to treatment | Encode at this node the portions of text that address the factors that complicate the communication with patients and caregivers regarding adherence to the prescription. |
| 5.1 Knowledge and skills for communication | Encode at this node the parts of the text that address the knowledge and skills needed to communicate with patients/caregivers regarding adherence to the prescription. |
| 5.1. Knowledge and skills they (health care workers) have | Encode at this node the parts of the text dealing with the knowledge and skills that health workers believe they have. |
| 5.2 Decision to communicate | Encode at this node the parts of the text that deal with deciding what to communicate to patients/caregivers. |
| 5.3 Factors that affect communication | Encode at this node the parts of the text dealing with the factors that affect the content and the way of communicating with the patients and caregivers. |
| 5.3.1 Time factor | Encode at this node the parts of the text dealing with the influence of the time factor on the content and the way of communicating with patient and caregivers. |
| 5.3.2 Availability factor | Encode at this node the parts of the text dealing with the influence of the availability of the drug on the content and the way of communicating with patients and caregivers. |
| 5.3.3 Money factor | Encode at this node the parts of the text dealing with the influence of the money factor on the content and the way of communicating. |
| 5.4 Most important factor | Encode at this node the parts of the text dealing with the most determining factor to ensure a good communication. |
| 5.4.1 Facilitating factors | Encode at this node the parts of the text dealing with the most determining factor to ensure a good communication, and how it facilitates the task. |
| 5.4.1 Complicating factor | Encode at this node the parts of the text dealing with the most determining factor to ensure a good communication, and how it complicates the task. |
| 5.5 The expectations of others that influence communication | Encode at this node the parts of text dealing with the expectations of others in the community that influence the content or modalities of communication. |

| Name | Description |
| --- | --- |
| 5.5. 1 Making it easy | Encode at this node the parts of the text that deal with how the expectations of others in the community influence the content or modalities, make the task easy. |
| 5.5.2 Complicating the task | Encode at this node the parts of the text that deal with how the expectations of others in the community influence content or modalities, complicate the task. |
| 5.6 The expectations of other medical professionals | Encode at this node the parts of texts that address the expectations of other medical professionals that influence the content or modalities of communication. |
| 5.6. 1. How it makes it easier | Encode at this node the parts of the text that deal with what might facilitate the task in relation to the expectations of other medical professionals who influence the content or modalities of the communication. |
| 5.6. 1. How it complicates the task | Encode at this node the parts of the text that deal with how the expectations of other medical professionals might complicate the communication with patients and caregivers. |
| 5.7 Impact of communication | Encode at this node the parts of the text dealing with the impact of communication with patients/caregivers on prescription adherence. |
| 5.8 Role of procedures and habits in communication | Encode at this node the portions of text that address the role that procedures and habits play in communicating with patients/caregivers regarding adherence to the prescription. |
| 5.8.1 What could facilitate | Encode at this node the parts of the text that deal with what could facilitate the role that procedures and habits play in communicating with patients/caregivers regarding adherence to the prescription |
| 5.8.2 What could complicate | Encode at this node the parts of the text that deal with what might complicate the role that procedures and habits play in communicating with patients/caregivers regarding adherence to the prescription |
| 5.9 Suggestions for a good communication | Encode at this node the parts of the text dealing with suggestions for good communication. |

Table 2: Support document of training and communication that was developed.

| **Topic** | **Behavior drivers** | **Details to include in the Training and Communication Package** |
| --- | --- | --- |
| Consultation | Fear | Reassure patients/caregivers that health care workers are there for them. |
|  | Stress related to the health condition of a loved one | Help the caregiver understand that the patient needs their cooperation and courage to recover. |
|  | Impatience of patients | - Explain to the patient that the time spent with the nurse is necessary to help him or her understand the explanation, follow the instructions, and get well. - Help the patient understand that there is no need to leave early if they need to return to the health center for the same disease. |
| The tests and results reporting |  | - Talk to the patient about all the types of samples that will be taken. - Reassure the patient about the need for these tests and about confidentiality. - Talk to the patient about possible outcomes. |
| Announcement of the diagnosis (if germs have been found) | Forgetfulness | - Ask the patient if he/she wants to involve someone else for explanations, then give the diagnosis. - Give all possible explanations about the disease(s) (name, mode of transmission,   evolution with or without treatment...) |
|  | Belief that some diseases cannot be treated at the health care center | - Help the patient to understand that despite the good will of the traditional healer to treat him, he does not have the tests or tools to provide a reliable diagnosis of his disease |
|  | Lack of  confidence in diagnosis | - Draw the patient's/caregiver's attention to the fact that nurses are well trained to care. |
| Announcement of Diagnosis (if no disease was found and no medication is needed) |  | - Explain to the patient why they do not need to take medication. - Show the patient why taking medication is not necessary in this case. |

| The prescription | Money | - Ensure that the medication prescribed is the most effective and least expensive. - Prescribe only what the patient needs after diagnosis (drugs recommended by the algorithm). - Control the availability of the drugs at the depot. - Reassure the caregiver that the care is free for children from 6 to 59 months, pregnant and postpartum women (42 days). - Convince the patient/caregiver of the need to start treatment immediately, and to complete it, and of the need to purchase the drug in a pharmaceutical depot. |
| --- | --- | --- |
|  | Some people think that injections are better than pills.  Some people think that low-cost drugs are not effective | - Explain and reassure the patient that the tablets will be effective for their disease and help them understand that generic drugs have the same molecules as specialty drugs. |

| Explanation of the prescription | The language barrier | - Ask another nurse for help; |
| --- | --- | --- |
|  | Lack of precision on the prescription (number of days)  Multiplicity of drugs | - Clearly and completely explain the prescription (doses per intake and per day, number of days) - Help patients/caregivers understand the need to take medications according to the number of days and the number of pills per dose and per day. |
|  | Misunderstanding of written instructions | - Provide patients/caregivers with signs to help them understand prescriptions. |
|  | Discontinuation of treatment in case of  improvement or no improvement | - Talk to the patient about side effects and give advice on how to deal with them if they occur. - Talk to the patient/caregiver about the need to finish the treatment even if they feel better. |
|  | Forgetfulness due to travel or field work; | - Ask the patient/caregiver to identify people who can help them remember to take or give the medication at the correct time. - Ask the patient/caregiver to repeat the explanations to the nurse. |
|  | Mother's sensitivity-  Non-  involvement of the father | − Ask the caregiver to involve the husband in administering the medication; |
|  | Education level; | - Ask the patient to relate the intake times to what is happening in the community (the sound of the school or church bell in the morning, at noon and at night). |

|  |  | - Use easy-to-understand language and give patients some signs to help them understand the prescriptions; |
| --- | --- | --- |
|  | Gender of the patient (shyness in  women) | - Reassure patients/caregivers that health care staff are there for them. |
| Scheduling of the follow-up | the distance between home and the health center | - Talk to the patient about the importance of follow-up.  - Ask the patient to begin to identify what she/he needs to be able to come to the follow-up from the first visit. |
|  |  |  |
